# Validation of four-dimensional flow cardiovascular magnetic resonance for aortic stenosis assessment

**DOI:** 10.1038/s41598-020-66659-6

**Published:** 2020-06-29

**Authors:** Gareth T. Archer, Alaa Elhawaz, Natasha Barker, Benjamin Fidock, Alexander Rothman, R. J. van der Geest, Rod Hose, Norman Briffa, Ian R. Hall, Ever Grech, Malenka Bissell, Abdallah Al-Mohammad, Thomas A. Treibel, Andrew J. Swift, James M. Wild, Pankaj Garg

**Affiliations:** 1 Department of Infection, Immunity & Cardiovascular Disease, University of Sheffield, Sheffield, UK; 2 Sheffield Teaching Hospitals NHS Foundation Trust, Sheffield, UK; 3 Division of Image Processing, Leiden University Medical Centre, Leiden, The Netherlands; 4 Division of Biomedical Imaging, Leeds Institute of Cardiovascular and Metabolic Medicine, University of Leeds, Leeds, UK; 5 Institute for Cardiovascular Sciences, University College London, London, UK; 6 Insigneo institute of in-silico medicine, University of Sheffield, Sheffield, United Kingdom

**Keywords:** Aortic diseases, Magnetic resonance imaging

## Abstract

The management of patients with aortic stenosis (AS) crucially depends on accurate diagnosis. The main aim of this study were to validate the four-dimensional flow (4D flow) cardiovascular magnetic resonance (CMR) methods for AS assessment. Eighteen patients with clinically severe AS were recruited. All patients had pre-valve intervention 6MWT, echocardiography and CMR with 4D flow. Of these, ten patients had a surgical valve replacement, and eight patients had successful transcatheter aortic valve implantation (TAVI). TAVI patients had invasive pressure gradient assessments. A repeat assessment was performed at 3–4 months to assess the remodelling response. The peak pressure gradient by 4D flow was comparable to an invasive pressure gradient (54 ± 26 mmHG vs 50 ± 34 mmHg, P = 0.67). However, Doppler yielded significantly higher pressure gradient compared to invasive assessment (61 ± 32 mmHG vs 50 ± 34 mmHg, P = 0.0002). 6MWT was associated with 4D flow CMR derived pressure gradient (r = −0.45, P = 0.01) and EOA (r = 0.54, P < 0.01) but only with Doppler EOA (r = 0.45, P = 0.01). Left ventricular mass regression was better associated with 4D flow derived pressure gradient change (r = 0.64, P = 0.04). 4D flow CMR offers an alternative method for non-invasive assessment of AS. In addition, 4D flow derived valve metrics have a superior association to prognostically relevant 6MWT and LV mass regression than echocardiography.

## Introduction

Aortic stenosis (AS) is the most common left-sided heart valve disease, and with an ageing population, the incidence is set to double over the next 20 years^1^. Once patients with severe AS develop symptoms and reduction in heart function, two-year mortality can reach 50% if the valve is not replaced. Timing of intervention depends on an accurate assessment of not only symptoms but also of AS severity. Transthoracic echocardiography (TTE) is the first-line test for the assessment of AS severity, left ventricular (LV) function and haemodynamics^2,3^. However, it is well-established that TTE has limitations – the approximation of blood flow as a single streamline by continuous-wave Doppler TTE overestimates valvular pressure gradients compared to invasive measurements^4,5^. This is because of the approximation of blood flow as a single streamline by continuous-wave Doppler TTE^6^. In addition, the effective orifice area (EOA) is calculated using the continuity equation which includes many geometric and physiological assumptions, in particular, the measurement of left ventricular outflow tract diameter is a significant source of error^7^. If there is diagnostic uncertainty or when there is a discrepancy between non-invasive and the clinical assessment of AS severity, guidelines recommend invasive cardiac catheterization for haemodynamic assessment in symptomatic patients^2^.

Cardiovascular magnetic resonance (CMR) imaging already offers a reference method for monitoring longitudinal changes in LV remodelling response in patients with AS^8^. Four-dimensional (4D) flow CMR is an emerging tool which allows quantifying cross-sectional x/y/z planner velocities over the complete cardiac cycle^9^. It has the advantage of identifying the true peak velocity across the three-dimensional aortic sinus and also circumvents many of the issues of echocardiographic measurement such as Doppler misalignment, as well as, flow and geometric assumptions. Being able to identify where the maximum velocity occurs in a three-dimensional (3D) space is a major advantage not only over Doppler TTE but also the current standard two-dimensional (2D) phase-contrast CMR methods for AS assessment – which is recognised to underestimate velocities^10,11^.

Moreover, it allows quantification of the EOA using the peak velocity plane, which coincides with the vena contracta, identified by an evaluation of the whole three-dimensional aortic sinus flow. However, there are many unknowns for wider adoption of these methods for AS assessment. Firstly, validation of peak velocity assessment by 4D flow CMR for estimating peak pressure drop across the aortic valve is lacking against the reference invasive method. Secondly, EOA calculation using the peak velocity plane (vena contracta) on 4D flow CMR has not been validated. Thirdly, it remains unclear if 4D flow CMR would offer any incremental benefit over Doppler TTE.

Thus, the main aims of this study were: (1) to validate the 4D flow CMR peak velocity assessment against the reference invasive pressure drop assessment, (2) to validate the 4D flow CMR velocity plane derived EOA against Doppler TTE derived EOA, (3) investigate if 4D flow CMR aortic valve assessment offers any better association to exercise tolerance evaluated by the six-minute walk test (6MWT) when compared to Doppler TTE, and (4) in the cohort with follow-up imaging studies, evaluate which measures are associated with LV remodelling.

## Methods

### Study population

This was a prospective, single-centre, sub-study of the EurValve programme (http://www.eurvalve.eu/). We recruited 18 patients with suspected severe aortic stenosis on echocardiography from the heart valve clinic. All patients who underwent 4D flow CMR prior to any valve intervention were also invited for post valve intervention follow-up 4D flow CMR and TTE at 3–4 months.

The inclusion criteria was clinically severe AS. The exclusion criteria were: moderate or severe aortic regurgitation, significant other valve diseases, coronary artery disease requiring coronary artery by-pass grafting surgery, limited pre- and post-intervention imaging data, any MRI contraindications or the inability to complete a six-minute walk test (6MWT).

### Ethics

This study was sponsored by the Sheffield Teaching Hospitals NHS Foundation Trust and approved by the National Research Ethics Service (17/LO/0283) in the UK. Written informed consent was obtained from all patients before participation. The study complied with the Declaration of Helsinki.

### Echocardiography

All echocardiograms were performed according to the British Society of Echocardiography guidelines for TTE examination^12^. Grading of aortic stenosis was performed as per the ESC guidelines using mean, and peak gradients and EOA was calculated by the continuity equation^2^. Patients received TTE examination before valve intervention (3–4 months prior to the invasive study), and a follow-up TTE was undertaken at 3–4 months.

### Invasive pressure gradient assessment

Invasive pressure gradients were obtained in all patients undergoing transcatheter aortic valve implantation (TAVI) as part of routine care prior to and post valve implantation. Cardiac catheterisation was performed using standard techniques via the femoral artery^13^. Seven-French pigtail catheters were placed in both the ascending aorta and the LV cavity, and simultaneous pressures were recorded^14^. The analysis was performed by the Xper CardioFlex system (Philips Healthcare, The Netherlands). Peak to peak pressure gradient was determined in millimetres of mercury (mmHg). This method is well established and has been used to define the natural history of AS and symptomatic development.

### CMR

CMR was performed on a 3 Tesla Philips Healthcare Ingenia system equipped with a 28-channel coil and Philips dStream digital broadband MR architecture technology. Patients received CMR examination before valve intervention (3–4 months prior to the invasive study) and a follow-up CMR study was done in 3–4 months.

### CMR protocol

The CMR protocol included a baseline survey, cines (vertical long axis, horizontal long axis, short‐axis contiguous left‐ventricle volume stack 3-chamber (LVOT-views) and aortic valve view cines). Cine images were acquired during end-expiratory breath-hold with a balanced steady-state free precession (bSSFP), single-slice breath-hold sequence. The number of LV short-axis slices varied according to the size of each patient’s heart.

Cine images had a spatial resolution of 2.5 × 2.5 mm^2^, interpolated to 1.56 × 1.56 mm^2^, and a slice thickness of 10 mm with contiguous slices for the short axis stack. Other imaging parameters were 30 phases, echo time (TE) = 1.5 ms, repetition time (TR) = 3.05 ms, flip angle = 45°, the field of view (FOV) was 400 mm, and SENSE factor 2–3.

### Four-dimensional flow CMR acquisition

For the 4D flow CMR acquisition, the initial VENC setting was estimated from TTE peak velocity and tested using a through-plane two-dimensional phase contrast acquisition. Further increments were added until aliasing disappeared across the aortic valve. Field-of-view was planned to cover the whole heart, aortic valve and ascending aorta. The 4D flow sequence used echo-planar imaging (EPI) acceleration factor of 5 with no respiratory gating. This sequence has been validated by previous studies for valvular flow quantification in humans at both 1.5 T and 3 T field strengths^15,16^. Other standard scan parameters were: acquired voxel size = 3×3×3 mm, reconstructed voxel size = 1.5×1.5×1.5 mm, echo time (TE) = 3.5 ms, repetition time (TR) = 10 ms, flip angle 10°, the FOV 340×340 and 30 cardiac phase.

Data pre-processing was done on the scanner to correct for phase offset errors such as eddy currents, Maxwell effects, and encoding errors related to gradient field distortions to avoid impairment of the measurements and inaccuracies in flow quantification^17,18^.

### CMR image analysis

All images were post-processed and analysed using offline research software called MASS (Version 2019 EXP, Leiden University Medical Centre, Leiden, The Netherlands). Left ventricular volumes, EF, and mass were calculated according to standard methods^19^.

### Four-dimensional flow CMR pressure gradient assessment

**Figure 1:**
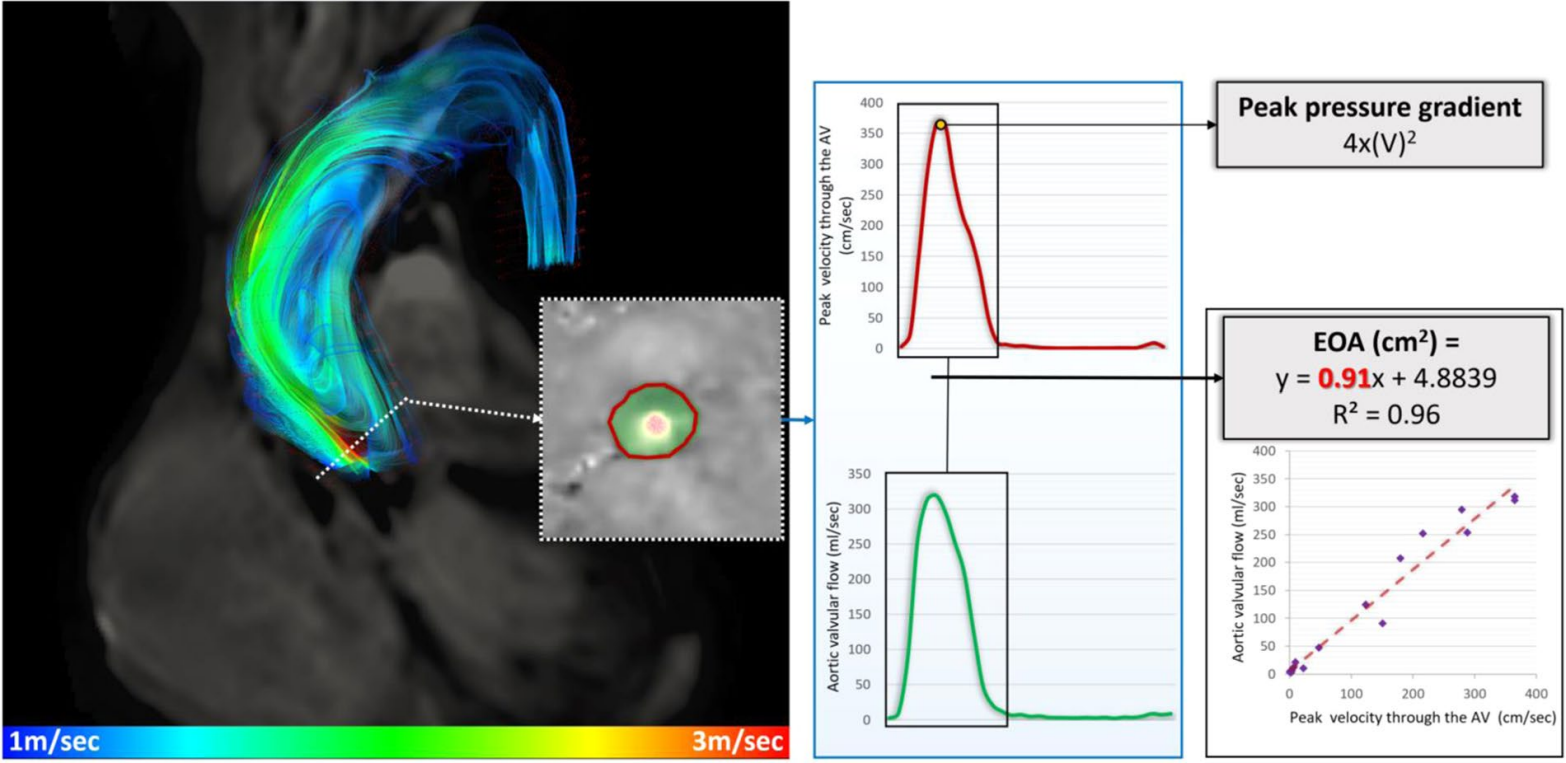
4D flow CMR for AS assessment. This figure is from a case example, which demonstrates how 4D flow CMR was used to investigate the peak velocity across the aortic valve. Using reformatted plane through the peak velocity plane, systolic flow and peak velocity curves were used to investigate the linear association. EOA is the gradient of the regression line.

All three-phase directions were screened for aliasing artefact, and if present, this was manually corrected using established phase unwrapping methods^20,21^. Any spatial misalignment with cine superimposition was manually corrected throughout the cardiac cycle prior to any quantification. The precise location of the maximum velocity (V_max_) in the aorta during systole was identified in the 4D flow data set and the velocity recorded in a similar method to Donati *et al*.^6^ (Fig. 1).

Steps taken to identify the peak trans-valvular velocity were as follows:

1. Firstly, a valvular plane was identified and tracked throughout the cardiac cycle (orange line in the supplementary video 1).

2. Several multi-planar slices 3/4 mm apart were done above the valve to assess the quality of the flow curves in the region of vena-contracta

3. The reformatted plane with the highest velocity and no artefact was selected. This was at the level of the vena contracta above the level of the valve.

Post valve intervention assessment is demonstrated in the supplementary online video. The maximum velocity determined in the 3D velocity data was used to determine the peak pressure drop by the simplified Bernoulli equation = 4(V_max_)^2^.

### Four-dimensional flow CMR effective orifice area (EOA) assessment

For EOA estimation, we applied the Bernoulli principles and the law of conservation of flow at the level of vena contracta across all systolic phases where the valve is maximally open. Time-resolved flow and velocity data were recorded, and as flow = area * velocity, EOA was estimated using the following equation:

$${\rm{EOA}}={{\rm{Flow}}}_{{\rm{t}}}/{{\rm{Vmax}}}_{{\rm{t}}}$$

Acceleration of the blood through the valve in early systole and the deceleration of blood prior to valve closure was recorded. An estimate of EOA was acquired using a line of best fit for the linear relationship of flow and the velocity at the vena contracta and calculating the gradient of that line (Fig. 1). Velocities at different flow rates throughout the systolic phases were recorded and used to reduce noise from the data, which may be higher if the EOA was calculated from one data point.

### Six-minute walk test

The six-minute walk test (6MWT) was carried out according to the guidelines outlined by the American Thoracic Society^22^. All tests were performed by the same clinician at the same location to avoid bias. None of the patients included had limiting arthropathy or airways disease.

### Statistical analysis

Statistical analysis was carried out with IBM SPSS Statistics version 25 software. Continuous measurements are presented as median with interquartile ranges (IQR). Normality of data was assessed by the Shapiro–Wilk test. Given the non-normal distribution of the data, a paired nonparametric two-tailed test (Wilcoxon signed-rank test) was used for paired analysis. Mann-Whitney test was used for all continuous variables to compare differences between two different procedure options of the aortic valve; for categorical variables, P-value was calculated using Chi-squared T-Test. Correlation between variables was assessed by Spearman correlation coefficient (rho), Value of P < 0.05 was considered significant. Bland Altman plots were used to assess the agreement of EOA by different methods. Comparison of variables amongst different New York Heart Association (NYHA) functional classes was performed using the Kruskal-Wallis H test. Furthermore, a Jonckheere-Terpstra test was carried out to find which specific groups of these independent variables are significantly different from each other. Results with a P-value of <0.05 were considered statistically significant.

## Results

### Demographic characteristics

Eighteen patients completed the full study protocol. Of these, eight patients underwent TAVI and ten patients surgical aortic valve replacement (SAVR). SAVR patients were younger than TAVI patients (68 ± 8 vs 82 ± 11, P = 0.01), and the 6MWT was better in SAVR patients than in TAVI (409 ± 182 meter vs 318 ± 96 meter, P = 0.02). A total of 6 post-operative patients (1 SAVR, 5 TAVI) declined to come back for research CMR scan. A full summary of the demographic data of the patients is shown in Table (1). Online Supplementary Table 1 provides detail on the type of replaced valve.

**Table 1:** Study demographics as per the final procedure the patient had.

	Patients chosen for TAVI (n = 8)		Patients chosen for SAVR (n = 10)		P
	Median	IQR	Median	IQR	
Age (years)	82	11	68	8	0.01
Sex (Female)	8	(100%)	6	(60%)	0.05
Height (cm)	1.6	0.035	1.7	0.13	0.08
Weight (Kg)	55.8	27.25	79.9	8	0.15
BMI (kg/m²)	23.35	9.75	28.1	4.4	0.32
SysBP (mmHg)	150.5	14	156.5	25	0.63
DiaBP (mmHg)	70.5	15	76	14	0.45
HR (bpm)	63.2	11	64.85	11	0.94
IHD	1	(12.50%)	0	(0%)	0.26
DM	2	(25%)	2	(20%)	0.81
Hypertension	7	(87.50%)	5	(50%)	0.10
Creatinine	68.5	20	79.5	26	0.25
ARB blocker	0	(0%)	1	(10%)	0.37
ACEi	2	(25%)	1	(10%)	0.41
Beta blocker	2	(25%)	2	(20%)	0.81
Ca channel blocker	3	(37.50%)	3	(30%)	0.74
Loop diuretics	3	(37.50%)	1	(10%)	0.18
Peak PG_TTE_ (mmHg)	77.6	27.5	64.6	22.3	0.12
Mean PG_TTE_ (mmHg)	40	11	32	8	0.22
6MWT (m)	318	96.5	409	182	0.02
NYHA	2	1	2	0	0.46

For all continuous variables, P-value was done using Mann-Whitney test.

For all categorical variables, P-value was calculated using chi-squared test.

### Invasive pressure gradient validation

**Figure 2:**
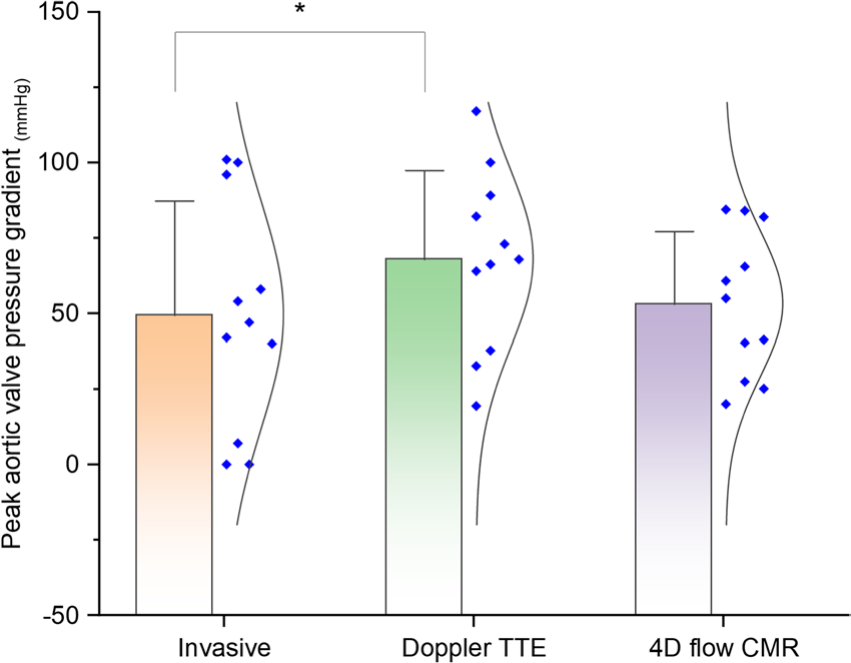
Histogram chart demonstrating the mean-plots of the peak pressure gradient across the aortic valve in cases that had measurements for all the three modalities - invasive, Doppler TTE and 4D flow CMR. *P < 0.05.

**Figure 3:**
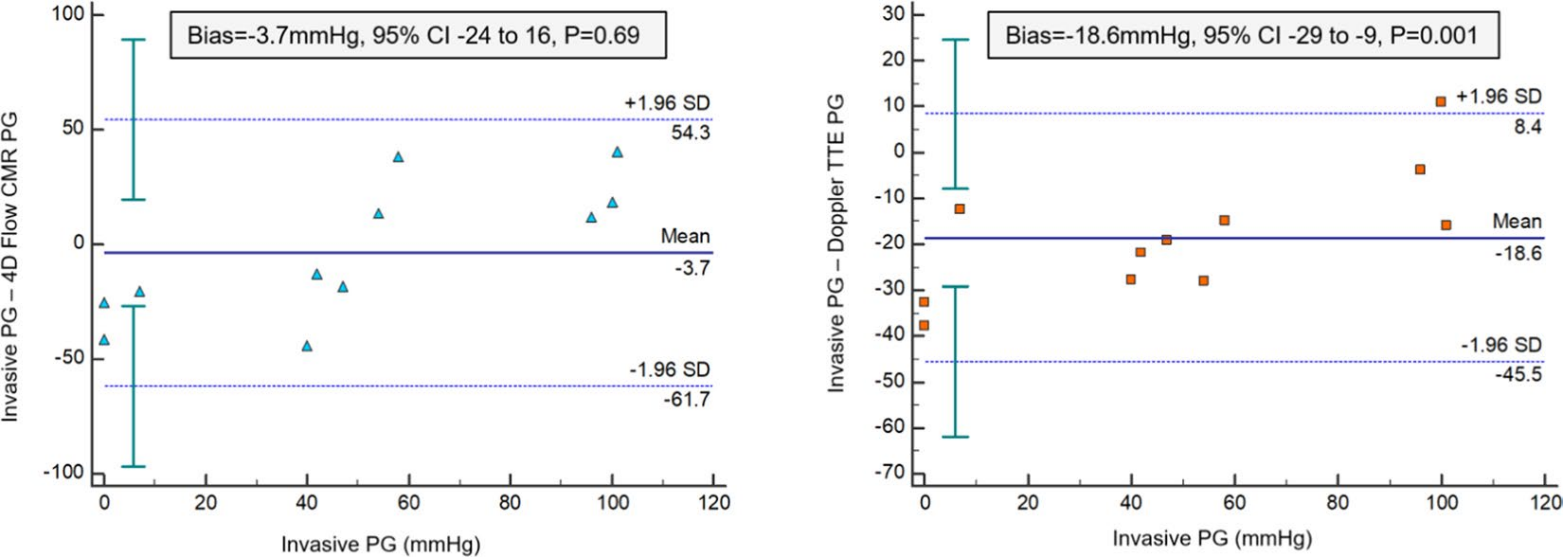
Bland-Altman plots for pressure gradients by 4D flow CMR and Doppler TTE against invasive study.

From the whole cohort, eight pre-intervention patients and three post-intervention patients received invasive catheter evaluation. The peak pressure gradient by 4D flow CMR was comparable to the invasive pressure gradient (54 ± 26 mmHG vs 50 ± 34 mmHg, P = 0.67). In contrast, Doppler TTE significantly overestimated the pressure gradient across the aortic valve when compared with invasive study (61 ± 32 mmHG vs 50 ± 34 mmHg, P = 0.0002) (Fig. 2). In addition, there was significant bias (−18.6 mmHg, P < 0.01) by Doppler TTE to estimate the peak pressure gradient (Fig. 3). Both Doppler TTE and 4D flow CMR derived pressure gradients demonstrated association with the corresponding invasive assessment (r = 0.95, P < 0.01; r = 0.63, P = 0.04). Using a cut-off of 64 mmHg peak pressure gradient for defining severe AS, the invasive assessment was more in agreement with 4D flow CMR (weighted Kappa = 0.25, 95% CI −0.39 to 0.89) than Doppler TTE (weighted Kappa = 0.16, 95% CI −0.16 to 0.47). Online Supplementary Table 2 details per patient recordings.

### EOA validation

**Figure 4:**
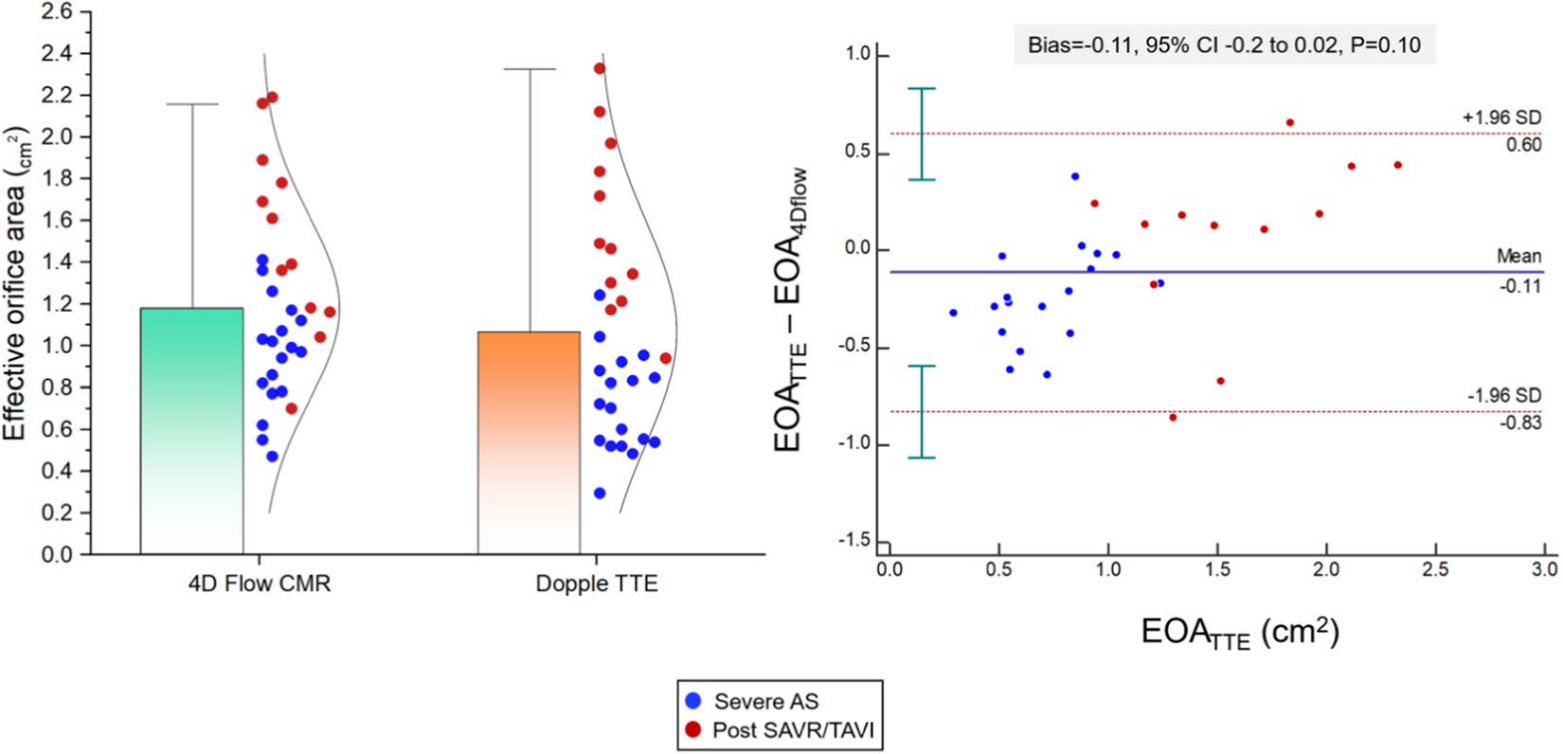
Mean and Bland-Altman plots for EOA between TTE and 4Dflow methods.

**Figure 5:**
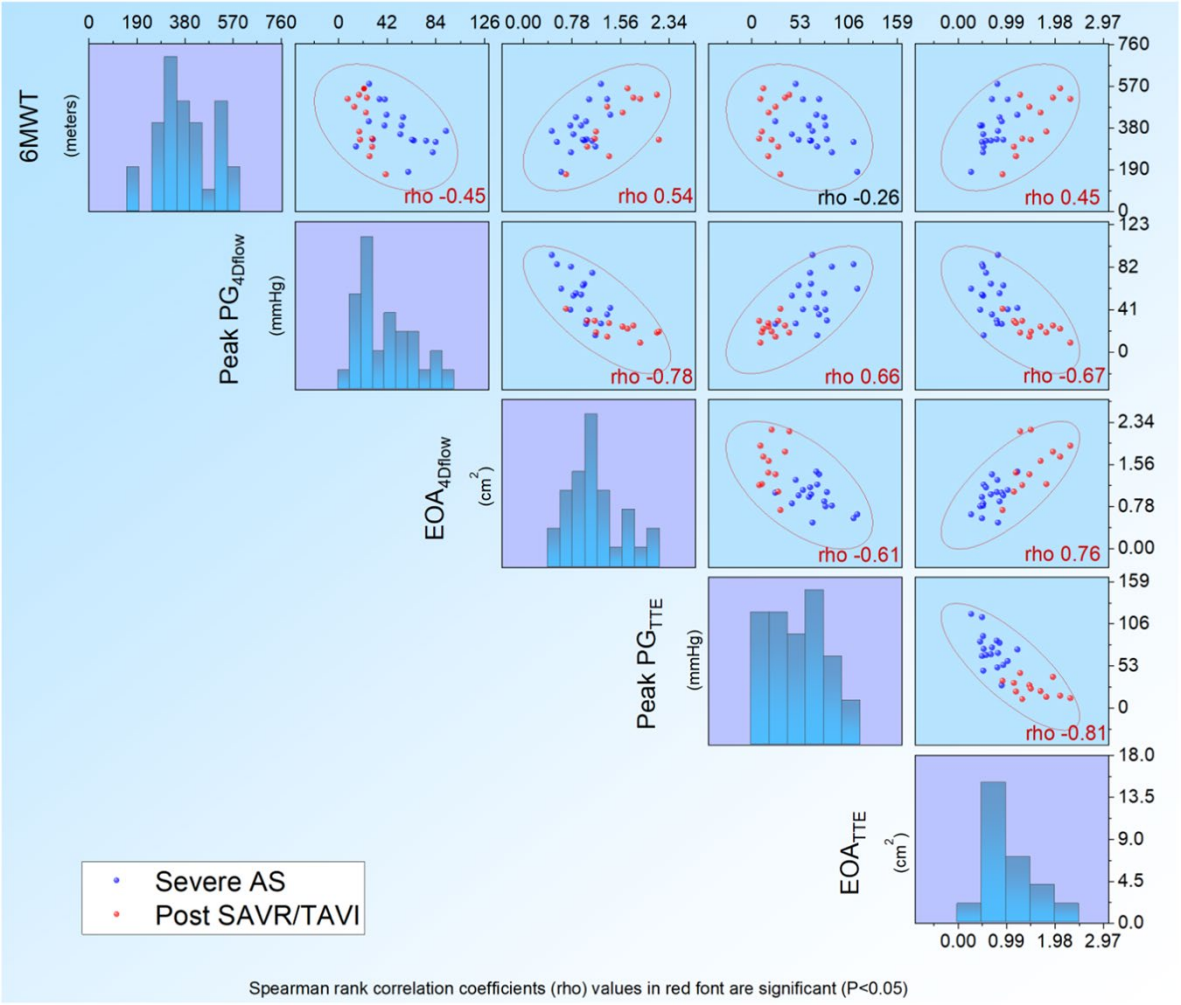
Correlation matrix summarises the association between the 6MWT and both TTE derived/4D flow CMR derive PG and EOA. 6MWT correlates with 4D derived pressure gradient and effective orifice area in patients with severe AS and post SAVR/TAVI. Both TTE and 4D flow CMR measurements demonstrate a correlation to each other.

Both 4Dflow and Doppler TTE derived EOAs were comparable (1.1 ± 0.5 cm^2^ versus 1.2 ± 0.4 cm^2^, P = 0.10, bias = −0.11, P = 0.10) (Fig. 4). In addition, the 4D flow derived EOA demonstrated a good correlation with Doppler TTE derived EOA (Fig. 5) for both pre-/post valve intervention cases.

### Association to 6MWT

There was a significant negative correlation observed between 6MWT and 4D flow CMR derived peak pressure gradient (r = −0.45, P = 0.01), 6MWT was also significantly associated with 4D flow CMR derived EOA (r = 0.54, P = 0.002) (Table 2, Fig. 5). However, the Doppler TTE derived peak pressure gradient did not demonstrate any significant correlation with 6MWT. Doppler-derived EOA showed good correlation to 6MWT (0.45, P = 0.01).

**Table 2:** Correlation between CMR derived metrics with both qualitative symptom burden (NYHA functional class) and exercise tolerance measured by the 6MWT.

	NYHA		6MWT	
	R*	P	R*	P
6MWT (m)	−0.099	0.60		
**Haemodynamic parameters**				
BP systolic (mmgH)	0.08	0.67	0.23	0.20
BP diastolic (mmgH)	0.18	0.34	0.26	0.14
HR (bpm)	−0.02	0.92	−0.27	0.22
**CMR functional parameters**				
LVEDV (mL)	0.15	0.45	0.36	0.05
LVESV (mL)	0.13	0.52	0.24	0.19
LV mass (g)	0.33	0.08	0.13	0.49
LV SV (mL)	0.14	0.46	0.36	0.05
MR EF (%)	−0.10	0.60	−0.23	0.22
**Aortic valve assessment**				
Peak PG_TTE_ (mmHg)	0.74	<0.01	−0.26	0.16
EOA_TTE_ (cm^2^)	−0.74	<0.01	0.45	0.02
Peak PG_4Dflow_ (mmHg)	0.56	<0.01	−0.45	0.02
EOA_4Dflow_ (cm^2^)	−0.51	<0.01	0.54	<0.01

*Spearman’s rho correlation coefficient.

### Association with NYHA functional status

There was no significant correlation between the NYHA classification and all parameters used in this study except for pressure gradient and EOA. Doppler TTE and 4D flow CMR pressure gradients were found to have a significant positive correlation with NYHA classification (r = 0.74, P < 0.001; r = 0.56, P = 0.001 respectively), Whereas, Doppler TTE and 4D flow CMR EOAs were found to be negatively associated with NYHA classification (r = −0.74, P < 0.001, r = −0.51, P = 0.003 respectively).

### Association with relative LV mass change

Spearman’s correlations were computed to determine if there were any significant relationships between the relative LV mass change and the relative change in other imaging parameters. The correlation appears to be statistically significant with the relative 4D flow CMR pressure gradient change pre/post valvular intervention (r = 0.64, P = 0.04), while TTE PG did not reach significance (r = 0.63, P = 0.06) (Table 3).

**Table 3:** Association of relative LV mass change to relative change in other imaging markers pre-/post aortic valve intervention.

	rho*	P
LV EF (%)	0.27	0.40
Peak PG_4Dflow_ (mmHg)	0.64	0.04
EOA_4Dflow_ (cm^2^)	0.25	0.45
Peak PG_TTE_ (mmHg)	0.56	0.06
EOA_TTE_ (cm^2^)	−0.08	0.79

The relative pressure gradient change pre/post valvular intervention, determined by 4D flow CMR correlated with the relative change of LV mass.

*Spearman’s rho correlation coefficient.

## Discussion

This is the first study to validate the peak pressure gradient across the aortic valve by 4D flow CMR against transthoracic echocardiography and the gold standard invasive method. In addition, we describe and validate a novel 4D flow CMR derived method for EOA measurement in pre-/post-aortic valve intervention against Doppler TTE. Importantly, we note that only for 4D flow CMR, both pressure gradient and EOA demonstrated association to exercise tolerance quantified by the 6MWT. Lastly, 4D flow CMR derived peak pressure gradient demonstrated association to LV mass regression at three-months.

### Pressure gradient assessment

Previous studies have demonstrated a discordance between the invasive and Doppler TTE peak pressure gradient assessment and that Doppler methods overestimate the peak pressure drop^7,23^. Many reasons for this overestimation have been proposed. Firstly, due to the inherent differences between Doppler pressure gradient method, which provides a maximum instantaneous pressure gradient at one-time point versus the invasive method that provides the peak-to-peak gradient which occurs at two different time points, can lead to this overestimation^24^. Secondly, if the gain setting on the Doppler scale is set high, it can lead to overestimation of peak velocity. Other reasons include human errors associated with the Doppler methods^25^. The 4D flow CMR methods described in this study also relies on the maximum instantaneous pressure gradient but did not result in any overestimation. In fact, for defining severe AS, 4D flow CMR derived pressure gradient was more consistent with the invasive method. Reduction in overestimation could be because the peak velocity plane was spatially identified by velocity vector visualisation. This technique is not routinely applied in Doppler TTE as peak velocity assessment is made by continuous-wave Doppler, which summates all velocities in one direction.

Similar to our study, previous work by Allen *et al*. have demonstrated a systematic bias between Doppler and 4D flow CMR for the assessment of peak velocity assessment in patients with AS^26^. They showed Doppler to overestimate peak velocities. On the contrary, Nordmeyer *et al*. have previously demonstrated that 4D flow assessment results in significantly higher peak transvalvular flow velocities (3.12 m/s versus 2.78 m/s, P < 0.05) in stenotic lesion when compared to Doppler^27^. However, the majority of patients in their study (56%) were with pulmonary stenosis, where Doppler alignment remains challenging. Furthermore, their patient population was different from our study. They mainly studied younger patients (26 ± 10 years old) with bi-cuspid aortic valve disease leading to complex eccentric jets in the aortic root, which are difficult to align by uni-directional encoded ultrasound imaging methods. Hence, it is more likely that ultrasound methods will underestimate true peak velocity in their study cohort. Similar to Nordmeyer *et al*. study, Gabbour *et al*. demonstrated that 4D flow resulted in significantly higher peak velocity than echocardiography in younger patients with various congenital heart diseases^28^. Both these studies imply that 4D flow derived peak velocity assessment is possibly superior to echocardiographic methods in complex aortic valve stenotic lesions. Our study provides complementary, supportive data that 4D flow derived peak pressure gradients across the aortic valve in mainly degenerative aortic stenosis is reliable and is in agreement with the invasive assessment.

### Effective orifice area assessment

EOA assessment offers complementary information when making a comprehensive assessment of AS. EOA is relatively pre-load independent when compared to peak velocity assessment. In addition, the novel EOA derived by 4D flow CMR described in this study is not subject to the geometric assumptions made by Doppler TTE. As this method is the gradient of the linear regression line between flow and velocity through the aortic valve, we speculate that it is still relevant in slow flow, low gradient aortic stenosis. Larger studies are needed to evaluate our proposed methods in these challenging cases of aortic stenosis.

### Severity of aortic stenosis and functional capacity

One of the most important clinical aspects to determine the timing of aortic valve intervention is symptom onset. This can be assessed subjectively by the NYHA functional class or more quantitatively by the 6MWT. More recently, studies have demonstrated that 6MWT predicts clinical outcomes and already, in some centres, the 6MWT is now part of the routine assessment for patients referred for TAVI^29^. In this study, it was noteworthy that it was only for 4D flow CMR, both pressure gradient and EOA were associated with both NYHA functional class and more importantly, with the 6MWT. A better association to the 6MWT may concur with enhanced prognostication for patients with aortic stenosis than Doppler TTE derived pressure gradients.

### Positive LV remodelling post intervention

It is well established that after aortic valve intervention, the LV mass regresses with decrease in afterload. LV mass regression is independently associated with improved long-term survival^30^. It is plausible to expect a proportionate decrease in afterload, or the pressure gradient across the aortic valve and LV mass post aortic valve replacement. In this study, LV mass regression demonstrated a slightly better correlation to 4D flow CMR derived pressure gradient change - again suggesting its superiority over the standard methods of assessment.

### Limitations

This study had several limitations. One key limitation is the small number of patients recruited to the study. At most, this study offers hypothesis-generating data for future larger studies, which are needed to further validate our findings. However, it is still plausible to conclude that 4D flow CMR offers an alternative non-invasive method to quantify AS and its severity. 4D flow CMR is currently not widely available and requires a significant acquisition and post-processing competence, but streamlining and simplification would facilitate clinical adoption. During the 4D flow acquisition, respiratory navigation was omitted, which may have had an impact on the accuracy of derived velocity parameters. However, studies that carried out a head-to-head comparison of whole-heart 4D flow CMR have demonstrated that for quantification of intra-cardiac flow, both respiratory navigated and non-respiratory navigated 4D flow CMR acquisitions are comparable^31^. Another limitation that could influence the quality of the velocity profile is a low temporal resolution (40 ms). Other confounding factors include variation in the heart rate and physiological conditions between the two acquisitions.

### Clinical perspective

Many of the standard methods used in the assessment of AS have been shown to have inherent inaccuracies^6,32^. This includes both the non-invasive Doppler TTE and invasive assessment. Importantly, discordance between EOA and the pressure gradient to grade the severity of AS can further the confusion. It is clinically desirable to have more non-invasive tools to reduce the clinical dilemma and make an affirmative diagnosis and grading of AS. This study demonstrates that the non-invasive, non-contrast 4D flow CMR can not only provide a clinically relevant measurement of pressure gradient and EOA, but also that these metrics have enhanced association with the prognostically relevant 6MWT in patients with AS. In this study, the accuracy of 4D flow CMR pressure gradient assessment was slightly better than Doppler TTE when compared against the reference invasive methods. However, the precision was slightly lower with 4D flow CMR pressure gradient. Hence, the results from this study suggest that in patients where Doppler TTE is inconsistent with symptoms and has discordant results, 4D flow CMR could help in clinical decision making for deciding on aortic valve intervention.

## Conclusion

4D flow CMR offers an alternative method for non-invasive assessment of aortic stenosis. In addition, 4D flow CMR derived valve metrics have a superior association to prognostically relevant 6MWT and LV mass regression than TTE. Future larger studies are warranted to investigate the clinical benefit of using 4D flow CMR derived AS severity to make clinical decisions.

### Ethics approval and consent to participate

This study was sponsored by the Sheffield Teaching Hospitals (STH) NHS Foundation Trust and approved by the National Research Ethics Service (17/LO/0283) in the UK.

### Consent for publication

Written informed consent was obtained from all patients before participation.

## Supplementary information

Supplementary Information.

Supplementary Information 2.

## Data Availability

Please contact author for data requests.
