# Supplementary material for: Validation of four-dimensional flow cardiovascular magnetic resonance for aortic stenosis assessment
Source: Sci Rep. 2020 Jun 29;10:10569. doi: 10.1038/s41598-020-66659-6 (PMC7324609; doi:10.1038/s41598-020-66659-6)
Supplement: Supplementary file 1 — Supplementary Information. [file 41598_2020_66659_MOESM1_ESM.docx]

**Validation of four-dimensional flow cardiovascular magnetic resonance for aortic stenosis assessment**

Gareth T Archer**^1,2^** (mda03gta@sheffield.ac.uk)**,** Alaa Elhawaz^1^ (afaelhawaz1@sheffield.ac.uk), Natasha Barker^1^ (nbarker1@sheffield.ac.uk), Benjamin Fidock^1^ (bfidock1@sheffield.ac.uk), Alexander Rothman^1^ (a.rothman@sheffield.ac.uk), R.J. van der Geest^3^ (R.J.van_der_Geest@lumc.nl), Rod Hose^1,6^ (d.r.hose@sheffield.ac.uk), Norman Briffa^1,2^ (n.briffa@nhs.net), Laurence O’Toole^2^ (laurence.o'toole@nhs.net), Ian R Hall^2^ (ian.hall15@nhs.net), Ever Grech^2^ (e.grech@nhs.net), Malenka Bissell^4^ (M.M.Bissell@leeds.ac.uk), Abdallah Al-Mohammad^2^ (abdallah.al-mohammad@nhs.net), Thomas A. Treibel (Thomas.Treibel@bartshealth.nhs.uk)^5^, Andrew J. Swift (a.j.swift@sheffield.ac.uk)^1.6^, James M. Wild^1,6^ (j.m.wild@sheffield.ac.uk), Pankaj Garg (P.Garg@sheffield.ac.uk)^1,6^

1 Department of Infection, Immunity & Cardiovascular Disease, University of Sheffield, Sheffield, UK

2 Sheffield Teaching Hospitals NHS Foundation Trust, Sheffield, UK

3 Division of Image Processing, Leiden University Medical Centre, Leiden, The Netherlands

4 Division of Biomedical Imaging, Leeds Institute of Cardiovascular and Metabolic Medicine, University of Leeds, Leeds, UK

5 Institute for Cardiovascular Sciences, University College London, London, UK.

6 Insigneo Institute of in-silico medicine, University of Sheffield

**Supplementary Table 1.** Type of valve patients received during aortic valve intervention (SAVR/TAVI).

| Patients | Type of valve implanted |
| --- | --- |
| Case 1 | Trifecta GT |
| Case 2 | Carpentier Edwards Perimount Magna Ease |
| Case 3 | Carpentier Edwards Perimount Magna Ease |
| Case 4 | Carpentier Edwards Perimount Magna Ease |
| Case 5 | Carbomedics Supra-Annular (Top Hat) |
| Case 6 | Livonova Bicarbon Bileaflet Slim Line |
| Case 7 | Sapien 3 Edwards TAVI |
| Case 8 | Medtronic CoreValve Evolut R |
| Case 9 | Livonova Bicarbon Bileaflet Slim |
| Case 10 | ON-X |
| Case 11 | ON-X |
| Case 12 | Medtronic CoreValve Evolut Pro R |

**Supplementary Table 2.** Peak pressure gradient recorded by all three modalities.

| **Case detail** | **Invasive (mmHg)** | **4D flow CMR (mmHg)** | **Doppler Echocardiography (mmHg)** |
| --- | --- | --- | --- |
| Pre-intervention | 101 | 61 | 117 |
| Pre-intervention | 100 | 82 | 89 |
| Pre-intervention | 96 | 84 | 100 |
| Pre-intervention | 58 | 20 | 73 |
| Pre-intervention | 54 | 40 | 82 |
| Pre-intervention | 47 | 66 | 66 |
| Pre-intervention | 42 | 55 | 64 |
| Pre-intervention | 40 | 84 | 68 |
| Post-TAVI | 7 | 27 | 19 |
| Post-TAVI | 0 | 41 | 33 |
| Post-TAVI | 0 | 25 | 38 |

**Supplementary Video 1.** A case of study patient who had transcutaneous aortic valve implantation (TAVI) with Edwards Sapien valve. Panel a | Three-chamber cine showing the placement of aortic valve prosthesis. Panel b | Demonstrates tracking of the aortic valve - the orange line is at the level of valvular annulus. Panel c | Demonstrates the velocity vector overlayed over the three-chamber cine. The peak velocity was noted two slices above the centre orange line. Panel d | Demonstrates reformated plane through the slices which show peak velocity. No significant artefacts were noted at this level of the reformated plane.
